# T cell exhaustion is associated with the risk of papillary thyroid carcinoma and can be a predictive and sensitive biomarker for diagnosis

**DOI:** 10.1186/s13000-021-01139-7

**Published:** 2021-08-31

**Authors:** Chumeng Zhu, Yuechu Dai, Hui Zhang, Yanyun Ruan, Yong Zhou, Yingjie Dai, Lilong Fan, Tianjun Jia, Hongsheng Lu, Qi Chen

**Affiliations:** 1 Precision Medicine Center, Taizhou Central Hospital (Taizhou University Hospital), Taizhou, 318000 Zhejiang People’s Republic of China; 2 College of Lab Medicine, Hebei North University, No.11, Zuanshi Road (south), Gaoxin District, Zhangjiakou, 075000 Hebei People’s Republic of China; 3 Department of Surgical Oncology, Taizhou Central Hospital (Taizhou University Hospital), Taizhou, 318000 Zhejiang, People’s Republic of China; 4 Department of Pathology, Taizhou Central Hospital (Taizhou University Hospital), Taizhou, 31800 Zhejiang, People’s Republic of China; 5 Clinical Laboratory, Taizhou Central Hospital (Taizhou University Hospital), Taizhou, 318000 Zhejiang, People’s Republic of China

**Keywords:** Papillary thyroid carcinoma, Nodular goiter, Hashimoto’s thyroiditis, Lymph node metastasis, PD-1, Biomarker

## Abstract

**Background:**

The incidence of papillary thyroid carcinoma (PTC) has been steadily increasing over the past decades. Hashimoto’s thyroiditis (HT) is the most common autoimmune disease, and is related to the pathogenesis of PTC. Programmed death-1 (PD-1) is currently used for the treatment of PTC, but there are very few studies on the clinical value of PD-1 in the diagnosis and targeted therapy of PTC.

**Methods:**

The expression of T, B, NK cells and PD-1 in the peripheral blood of 132 patients with PTC (PTC group), 48 patients with nodular goiter (NG group) and 63 healthy subjects (HP group) were detected by flow cytometry. The expression of plasma T3, T4, FT3, FT4, TSH, TGAb and TPO was detected by chemiluminescence immunoassay. Among 132 PTC, 49 PTC&HT and 83 PTC&noHT were included. Among 48 NG, 10 NG&HT and 38 NG&noHT were included. The expressions of programmed death- ligand1(PD-L1) in tumor tissues of PTC group and thyroid tissues of NG group, PD-1 and CD3 in tumor infiltration lymphocyte (TIL) were detected by immunohistochemistry.

**Results:**

The expression of FT3, TGAb, CD3^+^PD-1^+^, CD3^+^CD4^+^PD-1^+^ and CD3^+^CD8^+^PD-1^+^ in PTC and NG was significantly higher than that in the HP group. Moreover, CD3^+^PD-1^+^, CD3^+^CD4^+^PD-1^+^ and CD3^+^CD8^+^PD-1^+^ expression had significant differences between the PTC group and the NG group. In addition, the expression of TGAb, TPO, CD3^+^PD-1^+^, CD3^+^CD4^+^PD-1^+^ and CD3^+^CD8^+^PD-1^+^ in PTC&HT group was significantly higher than that in the PTC&noHT group. While, the expression of B cells, CD3^+^PD-1^+^, CD3^+^CD4^+^PD-1^+^ and CD3^+^CD8^+^PD-1^+^ in PTC&HT group was higher than that in NG&HT group. PD-1 showed a significant correlation with PTC lymph node metastasis. CD3^+^PD-1^+^ and CD3^+^CD4^+^PD-1^+^ was higher in N1 stage than in N0 stage. Immunohistochemical results showed that the expression of PD-1, CD3 and PD-L1 in PTC was significantly higher than that in NG.

**Conclusions:**

T cell exhaustion might act as a biomarker for the differential diagnosis of PTC and NG. Patients with PTC&HT have obvious T cell exhaustion and increased expression of PD-1, PD-L1.Targeting the PD-1/PD-L1 pathway could be a new approach to prevent malignant transformation from HT to PTC&HT in the future.

## Introduction

Thyroid cancer (TC) is the most common endocrine malignancy [1], and its incidence is steadily increasing over the past decades [2]. Papillary thyroid carcinoma (PTC) is the commonest subtype of TC [3]. Recent studies have confirmed that the annual increase in the incidence of TC is mainly due to the increase in the incidence of PTC, and is related to overdiagnosis [4]. The prognosis of PTC is good, however some patients are susceptible to lymph node metastasis, which is associated with poor prognosis. Few patients are insensitive to traditional surgery or develop postoperative recurrence. Therefore, it is important to discover new targets to improve the diagnosis and treatment of PTC. Immune cells play a vital role in the occurrence, development and prevention of tumors. Hashimoto’s thyroiditis (HT) is the most common autoimmune disease characterized by the destruction of thyroid cells by leukocytes and antibody-mediated immune processes. Compared with the normal population, HT patients have a higher probability of developing TC, but its pathogenesis remains unclear [5]. Some clinical studies have found that thyroid dysfunction may occur after use of PD-1/PD-L1 inhibitor [6]. In our previous study on the function of peripheral blood T cells in patients with papillary thyroid cancer, our team found that CD38 and HLA-DR molecules were increased in peripheral blood T cells of patients with papillary thyroid cancer and were associated with lymph node metastasis. It is suggested that there is immune imbalance in PTC patients and it is related to metastasis. Previous studies by other scholars have shown that, PD-L1 is related to the tumorigenesis and development of various cancers including PTC [7, 8]. In addition, Lubin [9] found that the expression of PD-L1 in papillary thyroid carcinoma caused by Hashimoto’s thyroiditis increased. The purpose of this study was to evaluate the level of T cell exhaustion in patients with PTC, determine whether PTC&HT can further promote the decline of T cell function and proliferation, and identify new diagnostic targets for PTC and PTC&HT. Targeting the PD-1/PD-L1 pathway may provide a new therapeutic strategy to treat PTC and prevent malignant transformation from HT to PTC&HT in the future.

## Material and methods

### Subjects

Peripheral blood samples were drawn from 132 PTC patients and 48 nodular goiter (NG) patients, all of whom underwent thyroidectomy. The diagnosis of nodular goiter and thyroid cancer after operation was through histopathology. The diagnosis of HT was based on the increase of TPO and TGAb in combination with histopathological features, clinical examination and ultrasound examination. The control group consisted of 63 patients from the physical examination group, except for NG and HT. All the patients were from Taizhou Central Hospital between June 2020 and January 2021. 132 tissue sections of PTC and 48 tissue sections of NG were confirmed by two professional pathologists in line with the current WHO classification system to diagnose PTC and metastatic lesions. People with other tumors or tumor history were excluded from this study. None of the patients received chemotherapy or radiotherapy. The PTC population included 107 females and 25 males, and their age ranged from 22 to 80 years. There was no significant difference in age and gender among the PTC, NG and HP groups (Table 1). In addition, the patients’ clinical stage, tumor stage, lymph node stage and extrathyroidal extension stage were listed according to the Tumor/Node/Metastasis (TNM) classification [10]. This study was approved by the Ethics Committee of Taizhou Central Hospital (Taizhou, PRC). Written formal consent was obtained from all subjects.

**Table 1:** Papillary thyroid carcinoma patients’ clinicopathological characteristics and laboratory data

	PTC	NG	HP	*P*	*P1*	*P2*	*P3*
n	132	48	63				
Age [year]	49[22 ~ 80]	52[29 ~ 71]	47[26 ~ 74]	0.06			
Gender				0.16			
Male	25	6	17				
Female	107	42	46				
T							
T1 + T2	130						
T3 + T4	2						
N							
N0	83						
N1a + N1b	49						
M							
M0	131						
M1	1						
Clinical stage							
I	122						
II	10						
Laboratory data							
T3[nmol/L]	1.18 [1.06,1.28]	1.23[1.12,1.31]	1.21 [1.12,1.31]	0.23			
T4[nmol/L]	8.20 [7.20,9.10]	8.40 [7.15,9.00]	8.00 [7.00,8.50]	0.22			
FT3[pmol/L]	3.36 ± 0.41	3.39 ± 0.35	3.22 ± 0.36	0.04*	0.03*	0.03*	0.61
FT4[pmol/L]	1.32 ± 0.19	1.34 ± 0.17	1.32 ± 0.15	0.69	0.92	0.44	0.43
TSH [μIU/mL]	1.55 [1.07,2.09]	1.64[0.99,2.80]	1.81[1.15,2.56]	0.24			
TGAb [IU/mL]	23.5[15.0,74.5]	21.0 [15.0,29.8]	16.0[15.0,28.0]	0.00*	0.00*	0.36	0.36
TPO [IU/mL]	34.0[28.0,61.0]	28.0[28.0,38.7]	38.0[28.0,55.0]	0.08			
CD3^+^ PD-1^+^ (%)	11.5 ± 4.7	5.9 ± 2.1	8.6 ± 4.5	0.00*	0.00*	0.00*	0.00*
CD3^+^CD4^+^PD-1^+^ (%)	7.9 ± 3.2	4.3 ± 1.8	6.43 ± 2.7	0.00*	0.00*	0.00*	0.00*
CD3^+^CD8^+^PD-1^+^ (%)	5.1 [2.3,6.0]	2.8 [1.7,3.3]	4.7[2.7,5.8]	0.00*	0.75	0.00*	0.00*

Data were expressed as mean ± standard deviation (SD), median [interquartile range]. The continuous variables were compared by using the Student’s t-test and the Mann-Whitney U test, the categorical variables were compared by using the χ2 or Fisher’s exact test between discovery and validation groups. *P*:statistical difference within each group, *P1*: PTC and HP, *P2*: TN and HP, *P3*: PTC and TN

* represents significant differences (*p* < 0.05)

### Sample acquisition

Venous blood samples were collected from all subjects in the morning after overnight fasting. 3 ml of peripheral blood was collected from PTC, NG patients and HP and added to EDTA-K2 anticoagulant-containing tubes. The T, B, NK cells and PD-1 were analyzed by flow cytometry (BD FACSAria II, USA). Then, the peripheral blood was centrifuged at 3000 rpm, and the upper layer of plasma was drawn. The plasma levels of T3, T4, FT3, FT4, TSH, TGAb and TPO (thyroid function indexes) were determined by chemiluminescence immunoassay (CLIA) with biochemical instruments (SIEMENS ADVIA Centaur XP, GER).

### Flow cytometric analysis

Peripheral blood and microspheres were incubated in the dark for 30 min, hemolysin was added to destroy the red blood cells, then the sample was centrifuged to add PBS and analyzed immediately using BD FACSAria II cytometer and FlowJo software (BD, USA). Finally, the percentages of total T, CD3^+^CD4^+^, CD3^+^CD8^+^ T cells, NK cells, B cells, CD4^+^HLA-DR^+^, CD8^+^HLA-DR^+^, CD8^+^CD38^+^ subsets, CD3^+^PD-1^+^, CD3^+^CD4^+^PD-1^+^ and CD3^+^CD8^+^PD-1^+^ were obtained. All the experiments were conducted according to the manufacturer’s instructions.

### Immunohistochemistry

Tissue sections were deparaffinized with xylene, then hydrated with alcohol, and antigen retrieval was performed by heating 0.01 sodium citrate buffer in a microwave oven for 15 min. The sections were quenched with 0.3% hydrogen peroxide for 10 min to block endogenous peroxidase activity. They were incubated with the monoclonal antibody against PD-L1 (E1L3N, 1:200; Cell Signaling Technology, USA),CD3(SP7, 1:500; MXB Biotechnology, PRC), PD-1(MX033, 1:500; MXB Biotechnology, PRC) overnight at 4 °C, then added anti-rabbit secondary antibodies (1:500; Pierce, Appleton, WI, USA) and incubated at room temperature for 20 min. After each treatment, washed 3 times with PBS for 3 min each time, and then developed with 3,3-diaminobenzidine. After the sections were counterstained with hematoxylin and differentiated with hydrochloric acid and alcohol, they were dehydrated, transparent, and fixed. Random fields were chosen for imaging, which were performed with Zeiss photomicroscope (Carl Zeiss Meditec, Dublin, CA, USA). Two pathologists reviewed immunohistochemistry staining results. Quantitative analysis of PD-L1 expression was conducted on the basis of staining intensity and extent(0, no staining; 1+, the total number of positive cells less than 25%; 2+, the total number of positive cells in 10–49%; and 3+, the total number of positive cells above 50%). At least 5–10 HPFS were randomly observed.

### Statistical analysis

All statistical analyses were conducted using SPSS v20.0 (IBM, USA) and GraphPad Prism8 (GraphPad Software, USA). If the data was normally distributed, mean ± standard deviation (SD) was used to express the data, and one-way analysis of variance and independent sample t-test was used to analyze the data. If the data was non-normally distributed, the median (Q25,Q75) was used to represent the data, and Kruskal-Wallis H test and Wilcoxon test was used to analyze the data. For data analysis between three groups, when one-way analysis of variance was used, further pairwise comparisons could be made irrespective of a significant difference. When the Kruskal-Wallis H test was used, further pairwise comparisons were made only when there was a significant difference between the three groups. A *p*-value < 0.05 was considered to be statistically significant.

## Results

### Clinicopathological characteristics and PD-1 expression in HP, NG and PTC groups

**Fig. 1:**
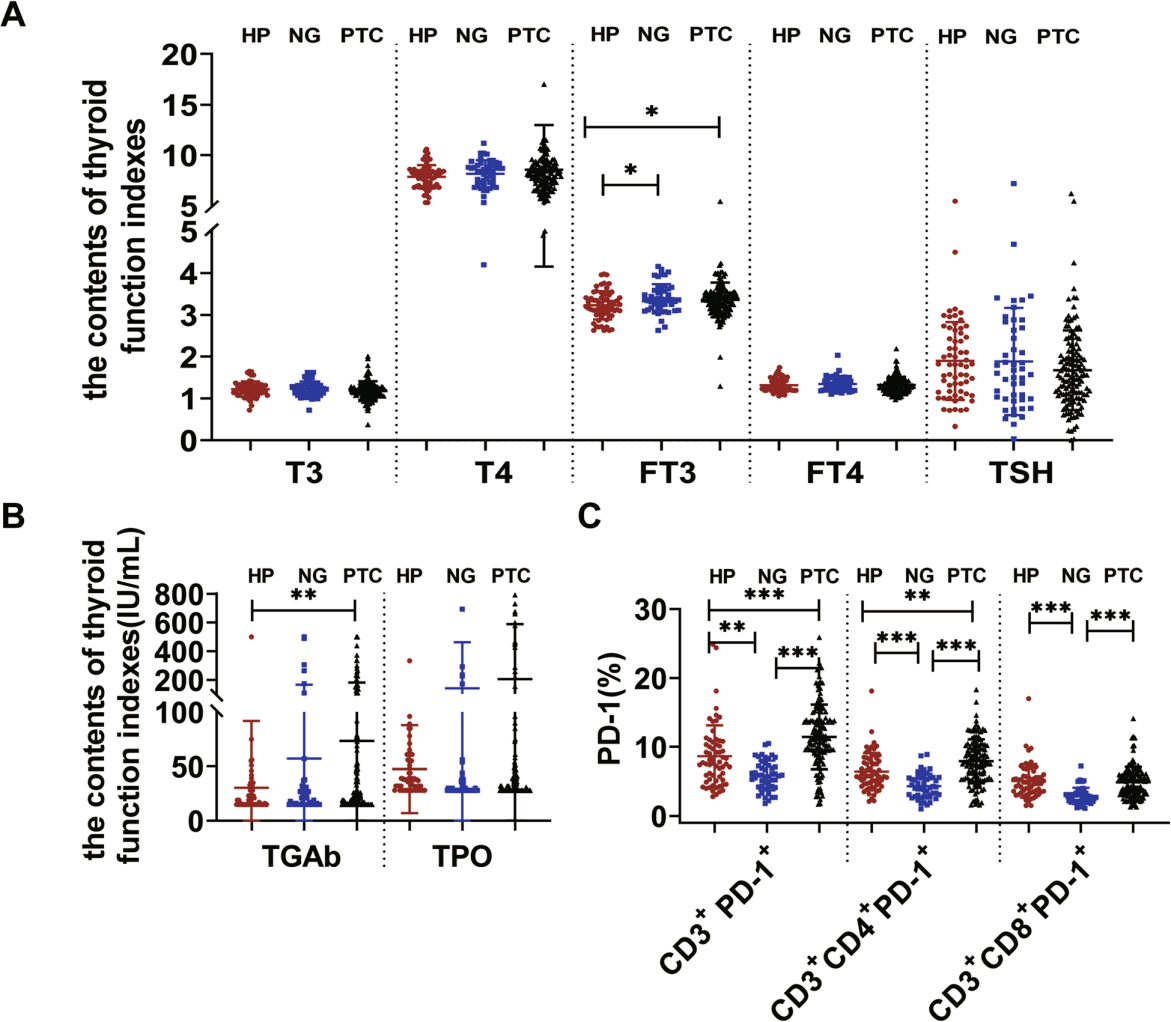
The activities of thyroid function indexes and PD-1. **A.** The plasma activities of the T3, T4, FT3, FT4, TSH among PTC, NG and HP groups. **B.** The plasma activities of the TGAb and TPO among PTC, NG and HP groups. **C.** The activities of the CD3^+^PD-1^+^, CD3^+^CD4^+^PD-1^+^ and CD3 + CD8 + PD-1+ among PTC, NG and HP groups (*P < 0.05), *P* < 0.05 represents a significant difference.

We examined the T3, T4, FT3, FT4, TSH, TGAb and TPO expression among the PTC group, NG group and HP group (Table 1, Fig. 1). The results showed that the expression of FT3 in the PTC group and NG group was significantly higher than that in the HP group, while the expression of TGAb in the PTC group was significantly higher than that in the HP group (*p* < 0.05). The expression of CD3^+^PD-1^+^ and CD3^+^CD4^+^PD-1^+^ was significantly higher in the PTC group than in the HP group, while the expression of CD3^+^ PD-1^+^, CD3^+^CD4^+^PD-1^+^ and CD3^+^CD8^+^PD-1^+^ was significantly lower in the NG group than in the HP group (*p* < 0.05) (Table 1, Fig. 1, Fig. 3). The expression of CD3^+^PD-1^+^, CD3^+^CD4^+^PD-1^+^ and CD3^+^CD8^+^PD-1^+^ in the PTC group was significantly different from that in the NG group (*p* < 0.05) (Table 1, Fig. 1).

### The expression of immune cells, thyroid function indexes and PD-1 in NG&HT, PTC&noHT and PTC&HT groups

**Fig. 2:**
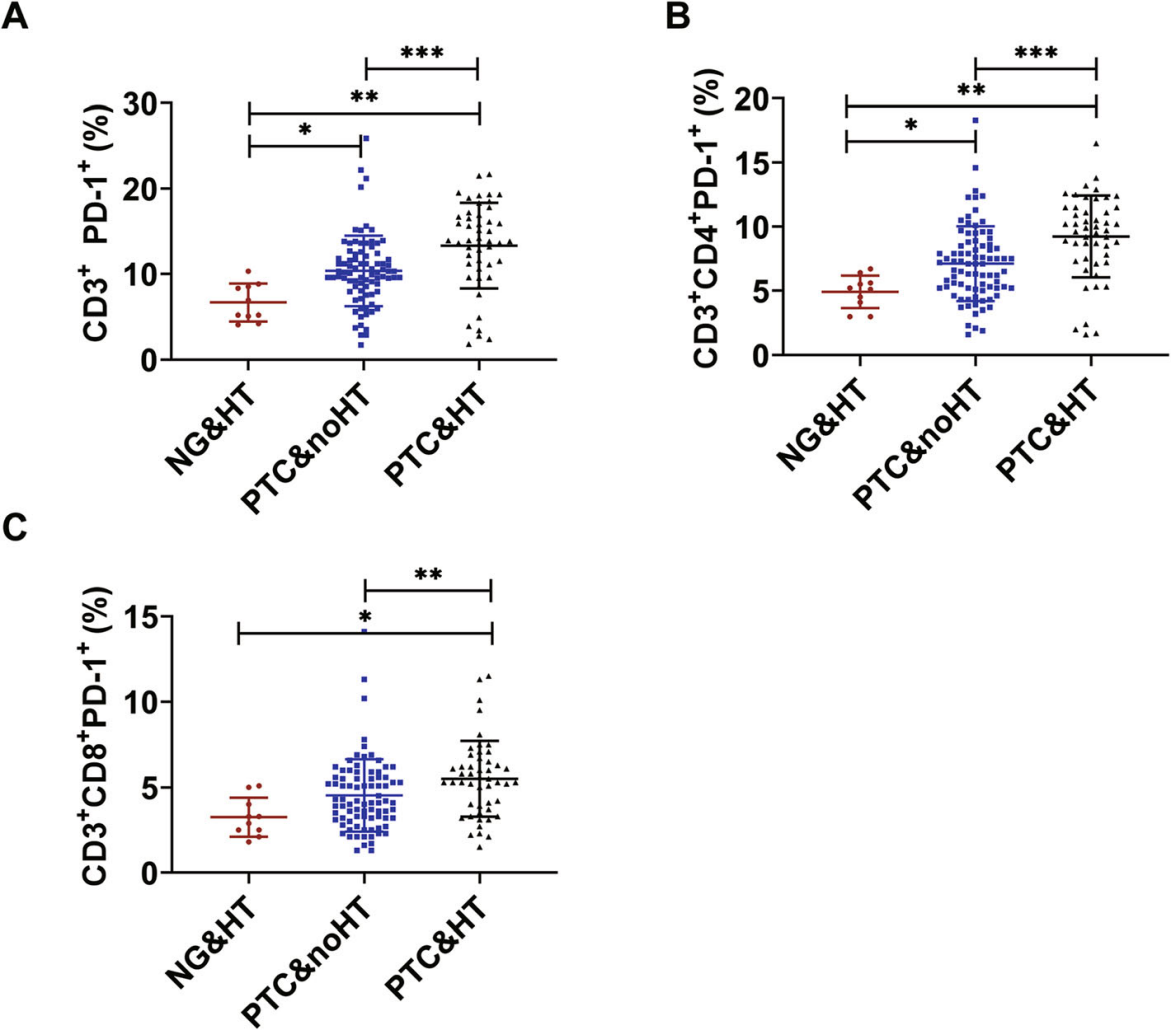
The activities of PD-1. **A.** The activities of the CD3 + PD-1+ among NG&HT, PTC&noHT and PTC&HT. **B.** The activities of the CD3 + CD4 + PD-1+ among NG&HT, PTC&noHT and PTC&HT. **C.** The activities of the CD3 + CD8 + PD-1+ among NG&HT, PTC&noHT and PTC&HT (*P < 0.05)

**Fig. 3:**
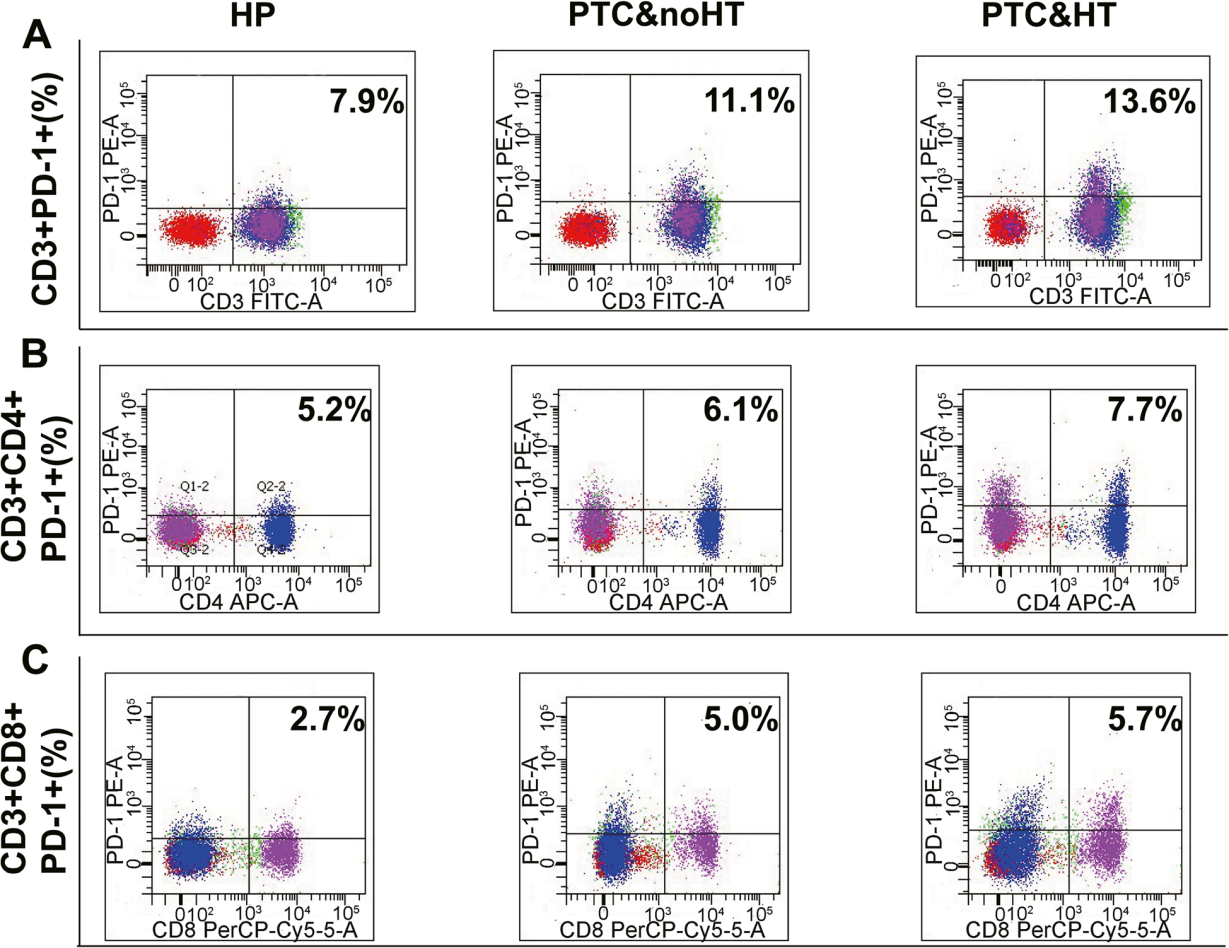
The expression of PD-1 in peripheral blood of HP, PTC&noHT and PTC&HT. **A.** The CD3 + PD-1+ expression among HP, PTC&noHT and PTC&HT. **B.** The CD3 + CD4 + PD-1+ expression among HP, PTC&noHT and PTC&HT. **C.** The CD3 + CD8 + PD-1+ expression among HP, PTC&noHT and PTC&HT(*P < 0.05)

To determine the differences in immune cells, thyroid function indexes and PD-1 between PTC&HT and PTC&noHT, we detected the expression of immune cells, thyroid function indexes and PD-1 among the two groups. The expression of TGAb, TPO, CD3^+^PD-1^+^, CD3^+^CD4^+^PD-1^+^ and CD3^+^CD8^+^PD-1^+^ was significantly higher in the PTC&HT group than in the PTC&noHT group (*p* < 0.05) (Table 2, Fig. 2, Fig. 3). In addition, no significant difference was observed in other T, B, NK cells, T3, T4, FT3, FT4 and TSH among the two groups (*p* > 0.05).

**Table 2:** The changes of lymphocyte subsets, thyroid function indexes and PD-1 among NG&HT, PTC&noHT and PTC&HT

	NG&HT (*n* = 10)	PTC&noHT(*n* = 83)	PTC&HT (*n* = 49)	*P*	*P*1	*P*2	*P*3
Laboratory Routine Data							
Total T(%)	60.92 ± 6.31	63.49 ± 8.49	62.38 ± 7.63	0.565	0.451	0.706	0.387
CD3^+^CD4^+^(%)	31.82 ± 3.39	37.24 ± 6.79	36.39 ± 5.93	0.132	0.047	0.093	0.523
CD3^+^CD8^+^(%)	26.10 ± 7.40	22.99 ± 7.40	22.56 ± 5.46	0.443	0.273	0.201	0.614
CD4^+^/CD8^+^(%)	1.21[0.90,1.78]	1.64 [1.19,2.37]	1.69 [1.25,2.18]	0.235			
CD4^+^ HLA-DR^+^(%)	7.37 ± 3.90	5.96 ± 2.06	6.34 ± 2.10	0.266	0.128	0.417	0.250
CD8^+^ HLA-DR^+^(%)	7.98 ± 3.96	6.6.5 ± 3.88	6.60 ± 2.84	0.633	0.376	0.341	0.834
CD8^+^CD38^+^(%)	8.96 ± 8.01	5.42 ± 2.81	5.70 ± 2.82	0.032*	0.009*	0.016*	0.694
NK(%)	23.05 ± 8.70	17.24 ± 8.20	18.23 ± 8.47	0.233	0.100	0.186	0.484
B(%)	10.85 ± 2.89	14.58 ± 5.10	15.33 ± 4.71	0.080	0.075	0.029*	0.281
T3(nmol/L)	1.21[1.15, 1.24]	1.20[1.07, 1.28]	1.16[1.06, 1.30]	0.472			
T4(nmol/L)	8.50[6.90,9.00]	8.20 [6.44,9.10]	8.20 [6.00,9.25]	0.991			
FT3(pmol/L)	3.28 ± 0.23	3.39 ± 0.37	3.32 ± 0.48	0.568	0.563	0.873	0.331
FT4(pmol/L)	1.29 ± 0.17	1.30 ± 0.18	1.35 ± 0.20	0.350	0.882	0.466	0.162
TSH (μIU/mL)	1.98[1.49, 4.69]	1.43[1.02, 1.94]	1.79[1.17, 2.46]	0.155			
TGAb (IU/mL)	218.0[29.0,489.0]	18.0[15.00,23.0]	125.0[33.0,243.5]	0.000*	0.000*	0.543	0.000*
TPO (IU/mL)	996.5[291.0,1300.0]	28.0[28.0,37.5]	258.0[36.0,909.0]	0.000*	0.001*	0.165	0.000*
CD3^+^ PD-1^+^ (%)	7.70 ± 2.10	10.35 ± 4.13	13.31 ± 5.04	0.000*	0.042*	0.006*	0.000*
CD3^+^CD4^+^PD-1^+^ (%)	4.97 ± 1.63	7.12 ± 2.93	9.24 ± 3.20	0.000*	0.049*	0.003*	0.000*
CD3^+^CD8^+^PD-1^+^ (%)	3.43 ± 1.35	4.53 ± 2.13	5.51 ± 2.22	0.012*	0.138	0.011*	0.005*
Immunohistochemistry							
TILS(%)	1(10.0%)	17(20.5%)	24(49.0%)	0.001*			
CD3 POSITIVE(%)	1(10.0%)	13(15.6%)	21(42.9%)	0.001*			
PD-1 POSITIVE(%)	0	12(14.5%)	14(28.6%)	0.038*			

*P*:statistical difference within each group, *P1*: NG&HT and PTC&noHT, *P2*: NG&HT and PTC&HT, *P3*: PTC&noHT and PTC&HT, * represents significant differences (*p* < 0.05)

In order to verify that T cell exhaustion in PTC was not completely caused by HT, we analyzed the expression of immune cells, thyroid function indexes and PD-1 in NG&HT group and PTC&HT group. The expression of B cell, CD3^+^PD-1^+^, CD3^+^CD4^+^PD-1^+^ and CD3^+^CD8^+^PD-1^+^ was significantly higher in the PTC&HT group than that in the NG&HT group (*p* < 0.05) (Table 2, Fig. 2).

### T cells infiltration (TIL) in PTC microenvironment

**Fig. 4:**
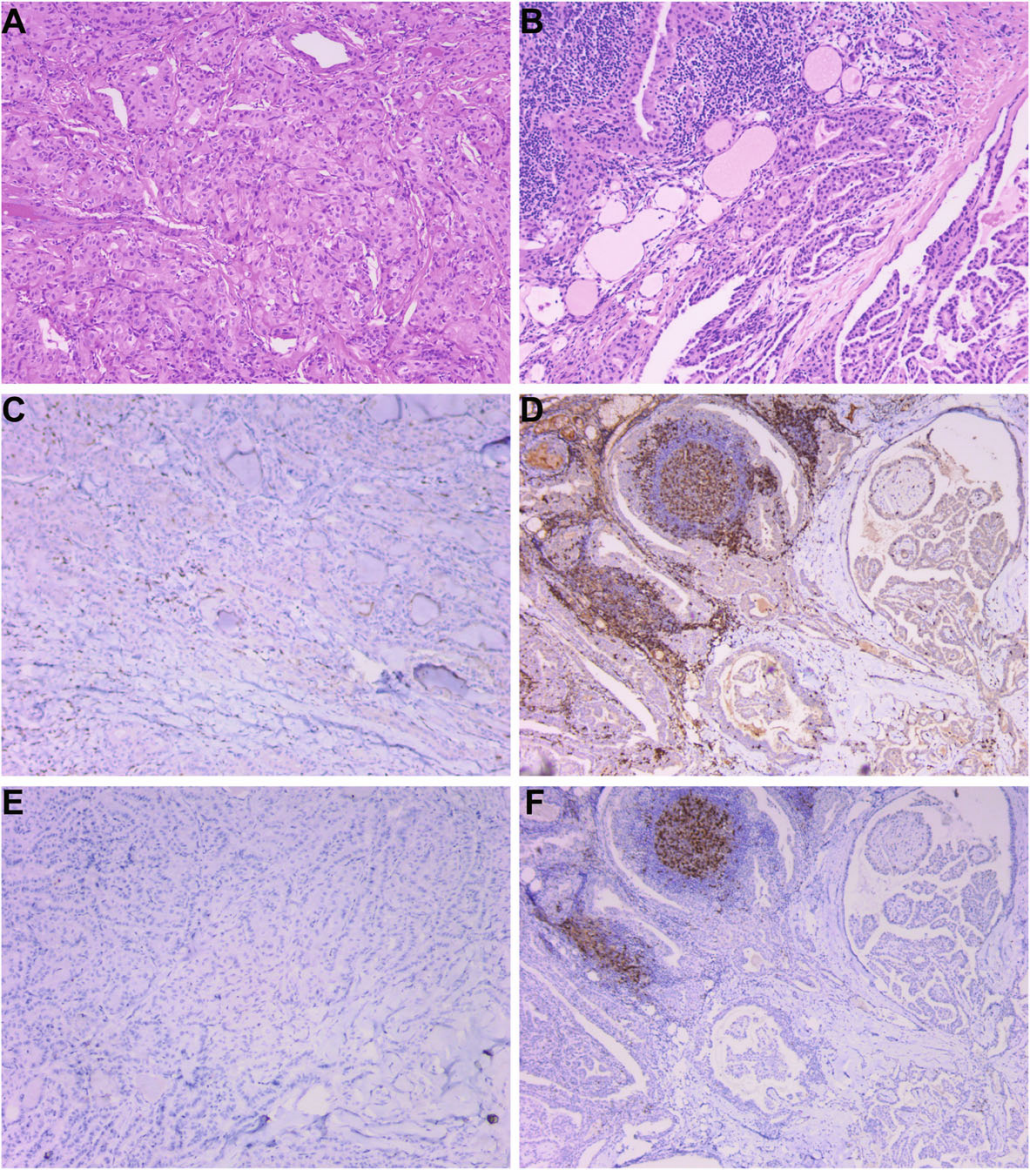
The T cells infiltration in thyroid by immunohistochemistry. **A.** PTC&noHT (100×). **B.** PTC&HT (100×). **C.** The CD3 expression in PTC&noHT (100×). **D.** The CD3 expression in PTC&HT (100×). **E.** The PD-1 expression in PTC&noHT (100×). **F.** The PD-1 expression in PTC&HT (100×)

To comfirm the correlation between immune cell fraction of peripheral blood and thyroid tumor microenvironment, immunohistochemical staining was performed in 10 NG&HT, 83 PTC&noHT and 49 PTC&HT tissue samples. The Positive proportions of TILS, CD3, PD-1 among the three groups were statistically significant. (Table 2, Fig. 4).

### The expression of PD-L1 in NG&noHT, NG&HT, PTC&noHT and PTC&HT tissues

**Fig. 5:**
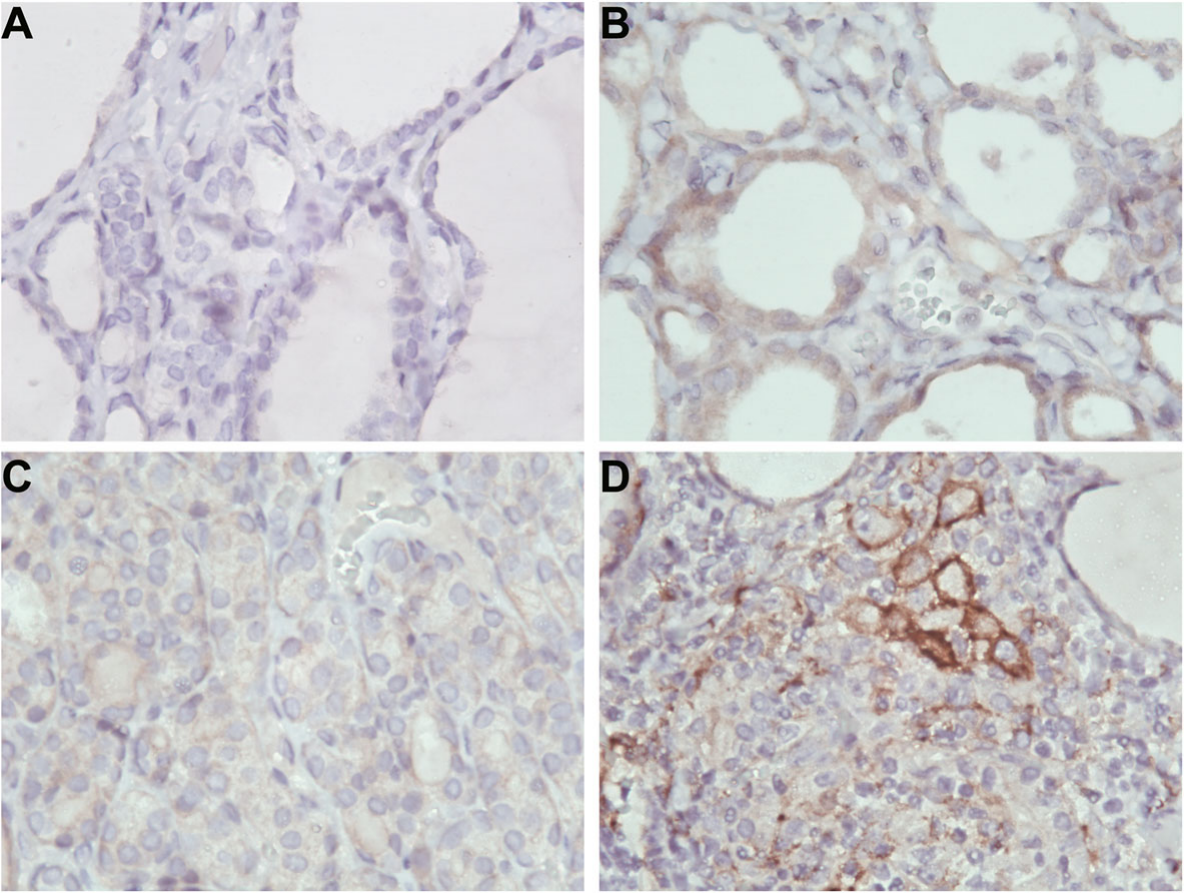
The expression of PD-L1 in NG&noHT, NG&HT, PTC&noHT and PTC&HT tissues. **A.** NG&noHT (400×). **B.** NG&HT (400×). **C.** PTC&noHT (400×). **D.** PTC&HT (400×)

**Fig. 6:**
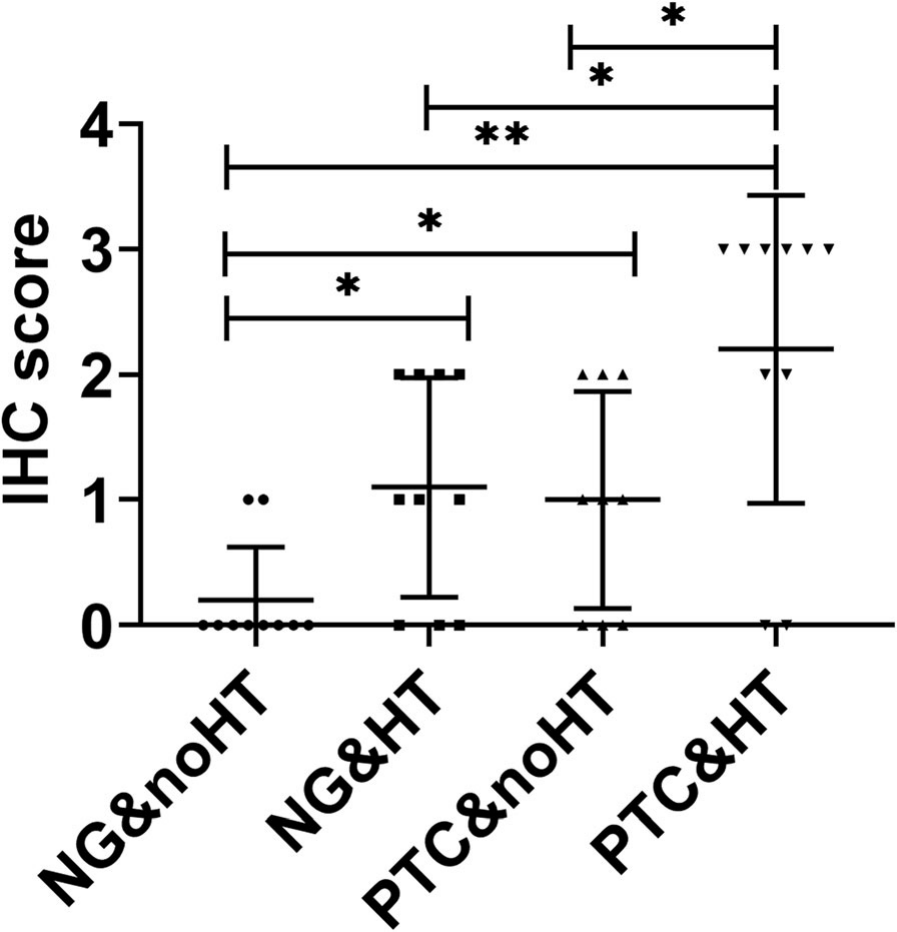
Immunohistochemical results of PD-L1 expression in NG&noHT, NG&HT, PTC&noHT and PTC&HT tissue samples. Immunopositivity scores were classified as follows: 3+ (a score of 3), 2+ (a score of 2), 1+ (a score of 1), and negative (a score of 0) (*P < 0.05)

To verify whether the status of T cells in tumor microenvironment was consistent with that in peripheral blood, immunohistochemical staining of PD-L1 was performed in 9 PTC&noHT, 10 NG&noHT, 10 NG&HT and 10 PTC&HT tissue samples. PD-L1 was positive in 2 NG&noHT (20%), 7 NG&HT (70%), 5 PTC&noHT (67%) and 8 PTC&HT (80%).The expression of PD-L1 in PTC&HT group was significantly higher than that in NG&HT group, the expression of PD-L1 in PTC&noHT group was significantly higher than that in NG&noHT group (*p* < 0.05) (Table 3, Fig. 5, Fig. 6).

**Table 3:** PD-L1 immunohistochemical staining results

	NG&noHT	NG&HT	PTC&noHT	PTC&HT
N	10	10	9	10
0	8	3	3	2
1+	2	3	3	0
2+	0	4	3	2
3+	0	0	0	6

### PD-1 expression was associated with PTC lymph node metastasis

**Fig. 7:**
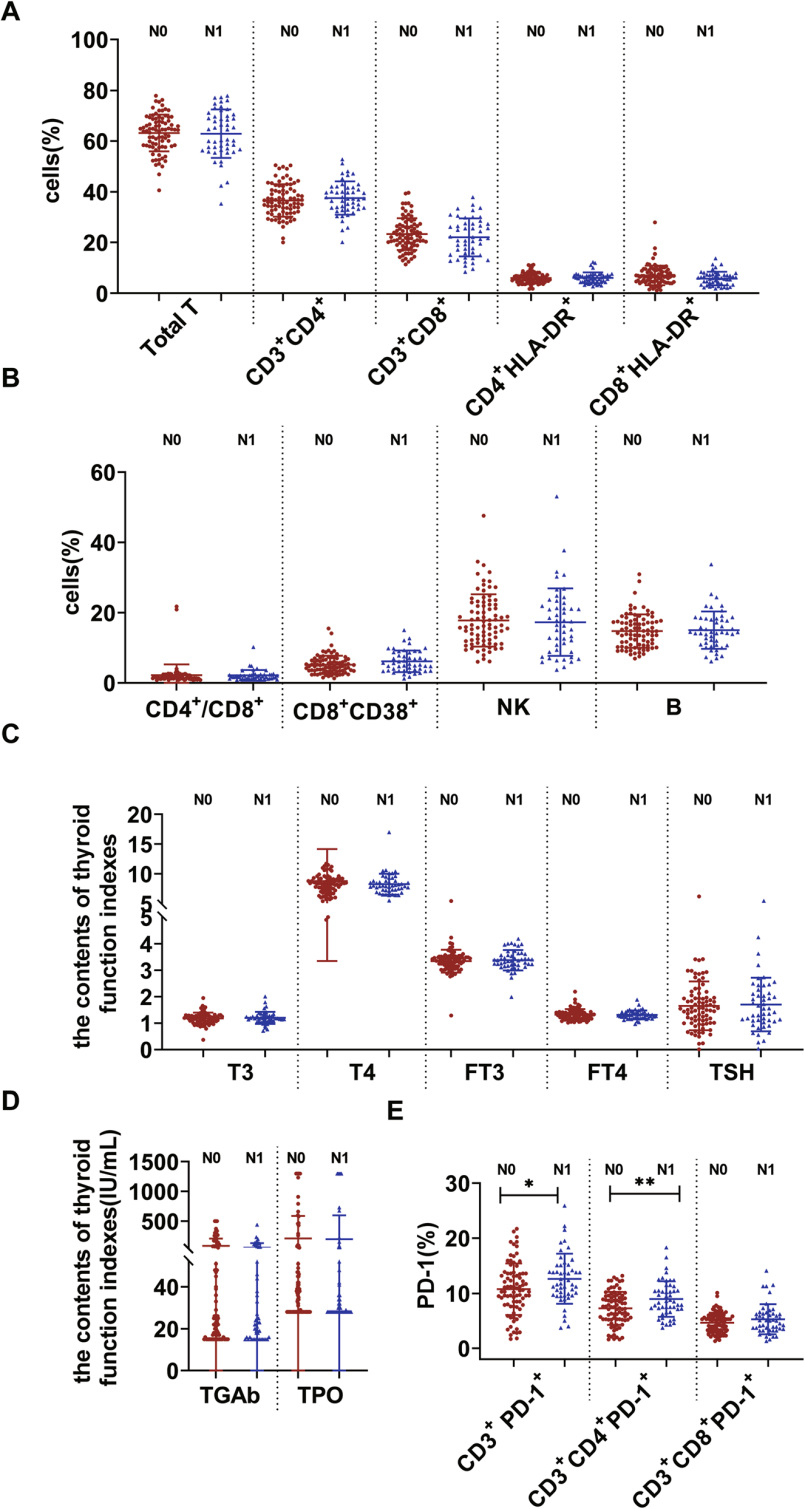
The activities of the NK cell, B cell, T cell subset, thyroid function indexes and PD-1. **A.** The activities of the Total T cells, CD3 + CD4+, CD3 + CD8+, CD4 + HLA-DR+ and CD8 + HLA-DR+ cells among N0 and N1. **B.** The activities of the CD4+/CD8+, CD8 + CD38+ cells, NK and B cells among N0 and N1. **C.** The plasma activities of the T3, T4, FT3, FT4, TSH among N0 and N1. **D.** The plasma activities of the TGAb and TPO among N0 and N1. **E.** The activities of the CD3 + PD-1+, CD3 + CD4 + PD-1+ and CD3 + CD8 + PD-1+ among N0 and N1(*P < 0.05)

To investigate whether PD-1, immune cells and thyroid function indexes are associated with PTC lymph node metastasis, we detected their expression levels. The expression of immune cells and thyroid function indexes showed no significant changes among the PTC N0 and PTC N1 stages (*p* > 0.05). In contrast, the expression of CD3^+^ PD-1^+^ and CD3^+^CD4^+^PD-1^+^ in the PTC N1 group was significantly higher than that in the N0 group (*p* < 0.05) (Table 4, Fig. 7). The above results indicated that PD-1 might be a novel prognostic indicator of PTC.

**Table 4:** Correlation between lymphocyte subsets, thyroid function index and lymph node metastasis of PTC patients

	N0 (*n* = 83)	N1 (*n* = 49)	*P*
Total T(%)	63.17 ± 7.26	62.92 ± 9.59	0.877
CD3^+^CD4^+^(%)	36.58 ± 6.41	37.50 ± 6.60	0.436
CD3^+^CD8^+^(%)	23.28 ± 6.27	22.07 ± 7.44	0.321
CD4^+^/CD8^+^(%)	1.64 [1.24,2.12]	1.76 [1.23,2.32]	0.382
CD4^+^ HLA-DR^+^(%)	6.06 ± 2.05	6.16 ± 2.14	0.802
CD8^+^ HLA-DR^+^(%)	6.70 [4.60,9.05]	6.00 [3.60,7.30]	0.059
CD8^+^CD38^+^(%)	4.80 [3.40,6.45]	5.40 [3.50,8.60]	0.084
NK(%)	17.81 ± 7.46	17.26 ± 9.59	0.715
B(%)	14.76 ± 4.75	15.01 ± 5.33	0.776
T3(nmol/L)	1.18 ± 0.22	1.20 ± 0.23	0.596
T4(nmol/L)	8.30 [7.15,9.15]	8.00 [7.30,8.80]	0.606
FT3(pmol/L)	3.38 [3.14,3.52]	3.37 [3.20,3.57]	0.655
FT4(pmol/L)	1.33 ± 0.20	1.31 ± 0.17	0.670
TSH (μIU/mL)	1.65 ± 0.92	1.71 ± 1.01	0.766
TGAb (IU/mL)	25.00 [15.50,78.00]	22.00 [15.00,52.00]	0.363
TPO (IU/mL)	37.00 [28.00,74.00]	30.00 [28.00,52.00]	0.188
CD3^+^ PD-1^+^ (%)	10.76 ± 4.68	12.63 ± 4.52	0.026*
CD3^+^CD4^+^PD-1^+^ (%)	7.27 ± 3.01	8.98 ± 3.24	0.003*
CD3^+^CD8^+^PD-1^+^ (%)	4.66 ± 1.81	5.29 ± 2.73	0.156

* represents significant differences (*p* < 0.05)

### The relationship between PD-1 and the diagnosis of PTC

**Fig. 8:**
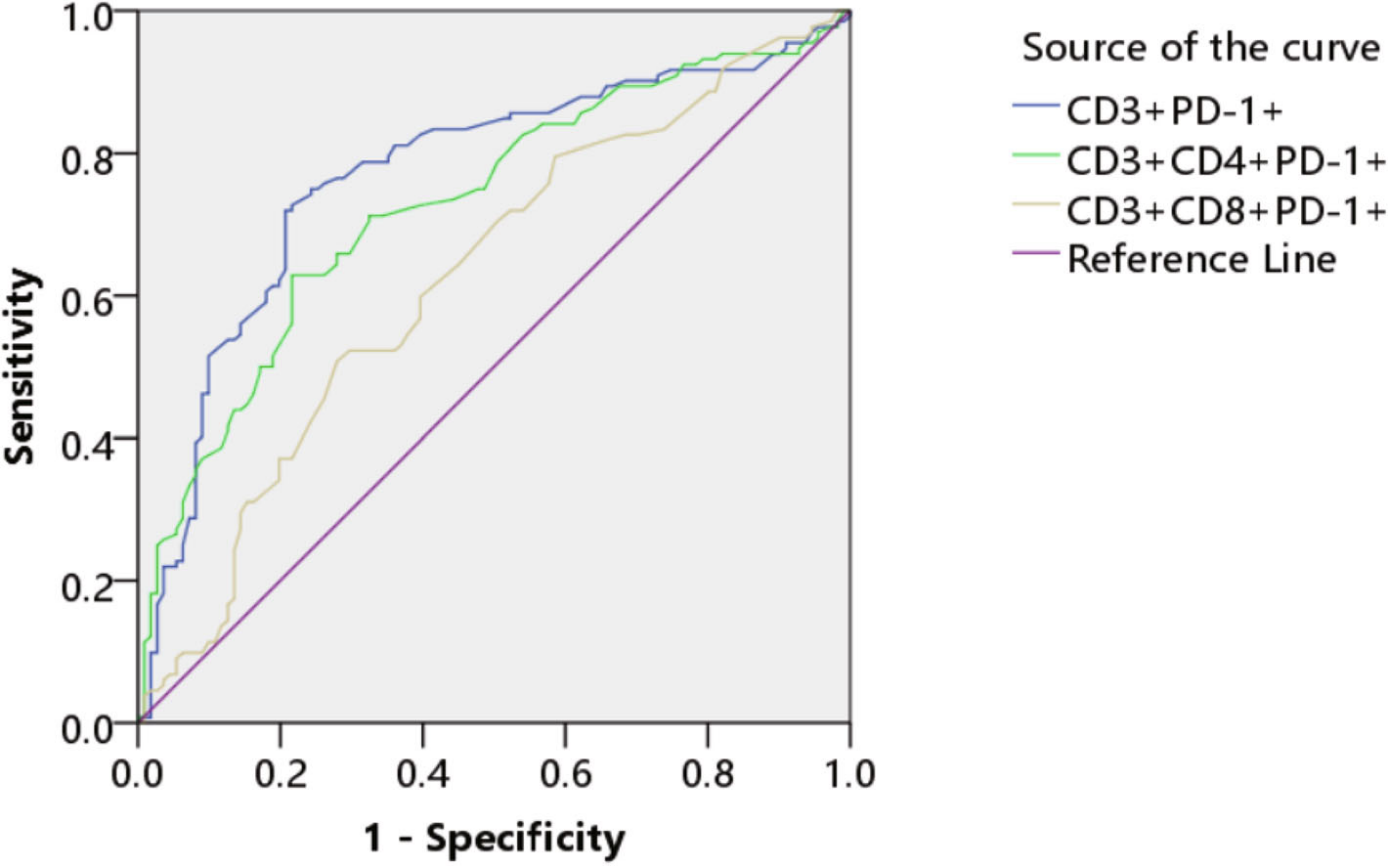
The ROC curve of CD3 + PD-1+, CD3 + CD4 + PD-1+ and CD3 + CD8 + PD-1 +

Using PTC clinical diagnosis as the gold standard, the ROC curves of CD3^+^PD-1^+^, CD3^+^CD4^+^PD-1^+^ and CD3^+^CD8^+^PD-1^+^ were drawn, with sensitivity as the ordinate and 1-specificity as the abscissa. The AUC values were compared. The AUC values were 0.770, 0,728 and 0.625, respectively (Fig. 8).

## Discussion

TC is the most common endocrine malignant tumor, with the fifth highest incidence among female tumors [2]. PTC accounts for 85% of all types of TC. Recent studies have shown that cancer cells can escape immune detection in the tumor microenvironment (TME). The normal microenvironment maintains tissue homeostasis and prevents the occurrence of tumors. Inflammatory mediators, reactive oxygen species, cytokines and chemokines from TME promote tumor growth. The relationship between TME and cancer cells is well established. The interaction between immune cells and cancer cells within TME contributes to all stages of cancer, starting from the early stage of tumorigenesis to the progression and metastasis of cancer [11, 12]. T cell immunity plays an important role in the immunosuppressive pathway. However, the immunosuppressive mechanism in TME weakens the effector function of T cells [13].

Antigen-specific T cells are the key protective factors against cancer, but continuous antigen stimulation leads to T cell exhaustion. The function and proliferation of exhausted T cells decreases, partly due to the overexpression of inhibitory receptors such as PD-1. Inflammation is associated with TC, which raises key questions about the role of immune cells in its pathogenesis. There are numerous immune cells and mediators in the TC ecosystem. Our previous study showed that CD8^+^CD38^+^T cells act as immune activation novel biomarkers and early warning indicator of patients with PTC [14]. PD-1 is highly expressed in activated T cells, B cells, dendritic cells and natural killer cells [15, 16]. Among immunosuppressive mechanisms, PD-1 has become the main marker of T cell dysfunction [7]. Many studies have shown that blocking the interaction between PD-1 and PD-L1 can reverse immune exhaustion and mediate anti-tumor activity [17–19]. PD-1 mainly restricts T cell activity in TME in the late stage of tumor growth [20]. PD-1 is activated after binding to PD-L1, and recruits phosphatase SHP-2 close to T cell receptor (TCR) and costimulatory signal to block the activation of PI3K and Akt mediated by CD28 [21]. The expression of PD-L1 and the status of cytotoxic T lymphocytes (CTL) in cancer patients are considered to be the decisive factors of their overall survival rate [22]. Blocking PD-1 pathway provides a new therapeutic approach for reinvigorating T cell response. In this study, the expression of CD3^+^ PD-1^+^ and CD3^+^CD4^+^ PD-1^+^ weas significantly increased in the NG and PTC groups, and the expression of PD-1 was significantly higher in PTC patients in N1 stage than those in N0 stage.

Many recent studies have confirmed the correlation between thyroid autoimmune diseases and the incidence of PTC. HT is the most common autoimmune disease characterized by the destruction of thyroid cells by leukocytes and antibody-mediated immune processes. It causes chronic inflammation of the thyroid tissue. Many studies have demonstrated the relationship between PTC and HT. Fiore et al. [23] found that the prevalence of PTC is higher in patients with nodular-HT than in patients with nodular goiter. Moreover, 5–10% of patients with both PTC and HT may develop aggressive disease and require systemic treatment [24]. Both TPO and TgAb are independent risk factors for TC. TPO is the enzyme responsible for the production of thyroid hormones. Li et al. [25] found that positive TGAb might be a risk factor for cervical lymph node metastasis. In this study, the expression of TPO and TGAb was significantly higher in the PTC&HT group than in the PTC group. However, there was no significant difference between thyroid function indexes and PTC lymph node metastasis. Meanwhile, we found that the expression of CD3^+^PD-1^+^, CD3^+^CD4^+^PD-1^+^ and CD3^+^CD8^+^PD-1^+^ was significantly higher in PTC&HT patients than in PTC without HT patients. In addition, to verify whether T cell exhaustion in PTC arise from HT, we analyzed the expression of immune cells and PD-1 in NG&HT and PTC&HT, and found that the expression of B cells, CD3^+^PD-1^+^, CD3^+^CD4^+^PD-1^+^ and CD3^+^CD8^+^PD-1^+^ in PTC&HT was significantly higher than that in NG&HT group. The results of immunohistochemistry also confirmed the above results of peripheral blood, and there were significant differences in the expression of PD-L1 between PTC and NG, as well as between PTC&HT and NG&HT. The above results suggested that PD-1 might act as an early warning indicator of the progression of HT patients to PTC.

In this study, we measured the expression of CD3^+^CD4^+^, CD3^+^CD8^+^ T cells, NK cells, B cells, CD4^+^HLA-DR^+^, CD8^+^HLA-DR^+^, CD8^+^CD38^+^, CD3^+^PD-1^+^, CD3^+^CD4^+^PD-1^+^ and CD3^+^CD8^+^PD-1^+^ by flow cytometry, and T3, T4, FT3, FT4, TSH, TGAb and TPO by CLIA among PTC group, NG group and HP group to determine whether they were related to the progression of PTC. We also explored the differences in CD3^+^CD4^+^, CD3^+^CD8^+^ T cells, NK cells, B cells, CD4^+^HLA-DR^+^, CD8^+^HLA-DR^+^, CD8^+^CD38^+^ subsets, CD3^+^PD-1^+^, CD3^+^CD4^+^ PD-1^+^ and CD3^+^CD8^+^PD-1^+^, T3, T4, FT3, FT4, TSH, TGAb and TPO expression between the PTC&HT group and the PTC group.

There are still some limitations in this research. This research is a retrospective study. Our data analysis was limited to clinical parameters and the exact geographic location of the samples collected. In addition, we did not carry out in vitro and in vivo experiments. It is planned to explore the key molecules and mechanism of HT to PTC in future research.

In summary, PTC has a certain degree of T cell exhaustion, especially complicated with HT. PD-1^+^T cells might act as a biomarker for the differential diagnosis of PTC and NG. PD-1 might be used as an early warning biomarker for the progression of HT to PTC. Targeting the PD-1 pathway could be a new approach to treat PTC and prevent malignant transformation from HT to PTC&HT in the future.

## Data Availability

The data used to support the findings of this study are included within the article.
